# Development and Validation of a Precise, Single HPLC Method for the Determination of Tolperisone Impurities in API and Pharmaceutical Dosage Forms

**DOI:** 10.3797/scipharm.1209-17

**Published:** 2012-11-05

**Authors:** Thummala Veera Raghava Raju, Raja Kumar Seshadri, Srinivas Arutla, Tharlapu Satya Sankarsana Jagan Mohan, Ivaturi Mrutyunjaya Rao, Someswara Rao Nittala

**Affiliations:** 1 Analytical Research and Development, Integrated Product Development, Dr. Reddy’s Laboratories Ltd., Bachupally, Hyderabad-500072, India.; 2 School of Chemistry, Andhra University, Visakhapatnam-530003, A.P., India.

**Keywords:** Tolperisone, Validation, Impurities, Degradation, HPLC

## Abstract

A novel, sensitive, stability-indicating HPLC method has been developed for the quantitative estimation of Tolperisone-related impurities in both bulk drugs and pharmaceutical dosage forms. Effective chromatographic separation was achieved on a C18 stationary phase with a simple mobile phase combination delivered in a simple gradient programme, and quantitation was by ultraviolet detection at 254 nm. The mobile phase consisted of a buffer and acetonitrile delivered at a flow rate 1.0 ml/min. The buffer consisted of 0.01 M potassium dihydrogen phosphate with the pH adjusted to 8.0 by using diethylamine. In the developed HPLC method, the resolution between Tolperisone and its four potential impurities was found to be greater than 2.0. Regression analysis showed an R value (correlation coefficient) of greater than 0.999 for the Tolperisone impurities. This method was capable of detecting all four impurities of Tolperisone at a level of 0.19 μg/mL with respect to the test concentration of 1000 μg/mL for a 10 µl injection volume. The tablets were subjected to the stress conditions of hydrolysis, oxidation, photolysis, and thermal degradation. Considerable degradation was found to occur in base hydrolysis, water hydrolysis, and oxidation. The stress samples were assayed against a qualified reference standard and the mass balance was found to be close to 100%. The established method was validated and found to be linear, accurate, precise, specific, robust, and rugged.

## Introduction

Impurity profiling of active pharmaceutical ingredients and formulations is one of the most challenging tasks of analytical chemists in the pharmaceutical industry [1]. The presence of unwanted or in certain cases unknown chemicals, even in small amounts, may influence not only the therapeutic efficacy but also the safety of the pharmaceutical products [2]. For these reasons, all major international pharmacopoeias have established maximum allowed limits for related compounds for both bulk and formulated APIs. As per the requirements of various regulatory authorities, the impurity profile study of drug substances and drug products has to be carried out using a suitable analytical method in the final product [3, 4].

**Fig. 1:**
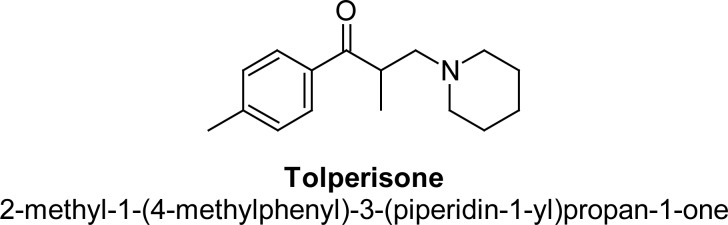
Structure of Tolperisone

Tolperisone (TOLP) [5], chemically 2-methyl-1-(4-methylphenyl)-3-(piperidin-3-yl)-propan-1-one, is a piperidine derivative [6]. It is a centrally-acting muscle relaxant [7, 8] which is used in the treatment of different pathological conditions like multilocular sclerosis, myelopathy, encephalomyelitis, Spondylosis, Spondylarthrosis, Cervical and lumbar syndromes, Arthrosis of the large joints, obliterating atherosclerosis of the extremity vessels, Diabetical angiopathy, Thromboangiitis obliterans, and Raynaud’s syndrome [9]. Its empirical formula is C_16_H_23_NO and structure is shown in Fig. 1.

**Fig. 2:**
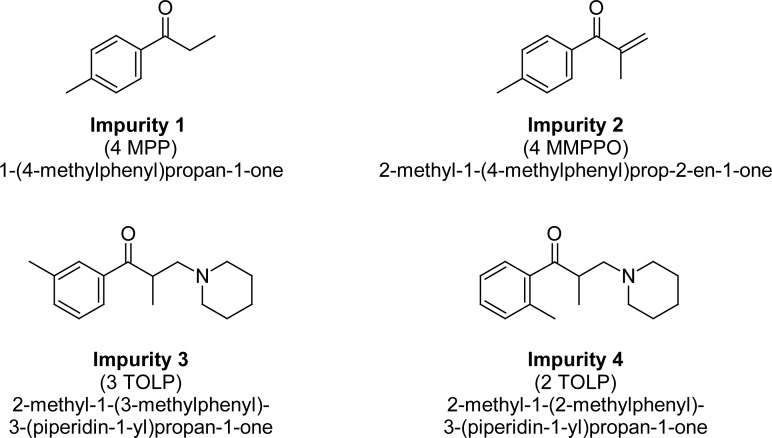
Structures of impurities

TOLP is a white to off-white crystalline powder with a molecular weight of 281.8. The route of synthesis of TOLP and possible degradants resulted in four known impurities, impurity 1, impurity 2, impurity 3, and impurity 4, which are not reported in any of the pharmacopeia. TOLP, impurities 3 & 4 are positional isomers. The structures of Tolperisone impurities are shown in Fig. 2. The presented method suffices the quantification of all known and unknown impurities of TOLP with more accuracy and precision.

TOLP is official in Japanese Pharmacopeia [10]. The literature survey revealed that there are some analytical methods reported for the estimation of TOLP either individually, like the visible spectrophotometric method, HPTLC, or in combination with other drugs by HPLC, and also reported on biological fluids [11–17]. There is not a single method that has been reported for the determination of the impurities, including positional isomers either in bulk drugs or in pharmaceutical formulations.

Hence, an attempt has been made to develop an accurate, rapid, specific, and reproducible method for the determination of TOLP impurities in bulk drug samples and in pharmaceutical dosage forms along with method validation as per ICH norms [18, 19]. The stability tests were also performed on both drug substances and drug products as per ICH norms [20, 21].

## Experimental

### Chemicals, reagents and samples

The active pharmaceutical ingredient in TOLP and its impurities were procured from the bulk manufacturers MS Sanochemia, Austria. The tablet dosage form was developed by Dr. Reddy’s, India. HPLC grade acetonitrile was purchased from Merck, Germany. Analytical reagents potassium dihydrogen orthophosphate, ortho phosphoric acid, diethyl amine, and sodium hydroxide were purchased from Merck, Germany. High purity water was prepared by using the Millipore Milli-Q plus purification system.

### Equipments

The Waters HPLC system with a diode array detector was used for method development, forced degradation studies, and method validation. The output signal was monitored and processed using Empower software. Photostability studies were carried out in a photostability chamber (SUN TEST XLS+, Atlas, USA). Thermal stability studies were performed in a dry air oven (Merck Pharmatech, Hyderabad, India).

### Chromatographic conditions

The chromatographic column Inert Sustine C18 (250 × 4.6) mm with 3 μm particles was used. The buffer consisted of 0.01 M potassium dihydrogen orthophosphate (pH adjusted to 8.0 with diethylamine). Mobile phase A consisted of a phosphate buffer and acetonitrile in the ratio of 50:50 (v/v). Mobile phase B consisted of a phosphate buffer and acetonitrile in the ratio of 30:70 (v/v). The flow rate of the mobile phase was 1.0 ml/min with a column temperature of 40°C and the detection wavelength at 254 nm. The gradient program was as follows: time (min)/%B; T_0.01_/50, T_5_/50, T_12_/60, T_15_/70, T_25_/90, T_30_/50, T_35_/50. The injection volume was 10 μl. The mixture of acetonitrile and 3.0 pH 0.01 M potassium dihydrogen orthophosphate in the ratio of 50:50 (v/v) was used as a diluent.

### Preparation of Standard and System Suitability Solution

A stock solution of TOLP (1000 μg/mL) was prepared by dissolving an appropriate amount in diluent. The working solution was prepared from the above stock solution for impurities’ determination (2 μg/mL of TOLP). A mixture of all impurities (2 μg/mL) along with TOLP (1000 μg/mL of TOLP) was also prepared in diluent.

### Preparation of Sample Solution

Twenty TOLP tablets (150 mg) were weighed and the average weight was calculated. The tablets were crushed into powder and the powder equivalent to 100 mg of the active pharmaceutical ingredient (TOLP) was transferred into a 100 ml volumetric flask. Approximately 70 ml of diluent was added, shaked to disperse the material, and sonicated for 30 minutes with intermediate shaking. The solution was then diluted to 100 ml and centrifuged at 3000 rpm for 10 min. The supernatant (1000 μg/mL of TOLP) was collected and filtered through a 0.45 μm pore size nylon 66-membrane filter. The filtrate was used as the sample solution.

## Method Validation

The method was validated for specificity, linearity, precision, accuracy, robustness, and ruggedness, according to ICH guidelines [19].

### System suitability

Having optimized the efficiency of a chromatographic separation, the quality of the chromatography was monitored by applying the following system suitability tests: resolution, asymmetric factor, and theoretical plates. The system suitability method acceptance criteria set in each chromatogram of standard solution were: resolution between any of the components > 2.0, tailing factor ≤2.0, and theoretical plates >2500.

### Specificity

Specificity is the ability of the method to measure the analyte response in the presence of its potential impurities. The specificity of the developed LC method for TOLP was carried out in the presence of all four impurities. Stress studies were performed at an initial concentration of 1000 μg/mL of TOLP in the active pharmaceutical ingredients (API) and formulated the sample to provide the stability-indicating property and specificity of the proposed method. Intentional degradation was attempted by the stress conditions of photolytic (exposed to UV light 200 watt hours followed by visible light 1.2 million lux), heat (exposed at 105°C for 2 hrs), acid (0.1N HCl refluxed for 1 hr at 60°C), base (0.1N NaOH refluxed for 45min at 60°C), oxidation (8% H_2_O_2_ refluxed for 45 min at 60°C), water (refluxed for 90 min at 60°C), and humidity (exposed to sat. KNO_3_ for 10 days).

### Precision

The precision of the impurities’ method was checked by injecting six individual preparations of (1000 μg/mL) TOLP spiked with 0.5% of all impurities. The RSD of the % individual impurity from six preparations was calculated.

The intermediate precision (Ruggedness) of the method was also evaluated by a different analyst, different column, and different instrument in the same laboratory.

### Limit of detection (LOD) and limit of quantification (LOQ)

The detection limit of an individual analytical procedure is the lowest amount of analyte in a sample which can be detected, but not necessarily quantitated as an exact value. The signal-to-noise ratio was determined by comparing the peak heights of the known concentrations of each related substance with that of the baseline noise obtained from the blank samples. A signal-to-noise ratio between 2.0 to 3.4 is generally considered acceptable for estimating the detection limit. The detection limits of individual compounds were different due to their peak shapes, retention time, and extinction coefficient. Limit of quantification is the minimum concentration at which the analyte can be reliably quantified with acceptable accuracy and precision, where the determination of the signal-to-noise ratio is performed by comparing measured signals from samples with known low concentrations of analyte with those of blank samples. A typical signal-to-noise ratio of 9.0 to 11.4 is generally considered acceptable for estimating the quantification limit. The LOD and LOQ for TOLP impurities were estimated, the precision and accuracy were also determined at the LOQ level, and the % RSD and recovery was calculated for the peak area for each impurity and for the TOLP.

### Linearity

The detector response for all four impurities was assessed by injecting six separately prepared solutions covering the range of the LOQ to 200% of the normal limit concentration. The correlation coefficients, slopes, and Y-intercepts of the calibration curve were studied.

### Accuracy

The accuracy of an analytical procedure expresses the closeness of agreement between the true value and the observed value. The study was carried out in triplicate by spiking impurities on the test preparation at 0.05%, 0.25%, 0.5%, 0.75%, and 1.0% of the analyte concentration (1000 μg/mL). The percentage of recoveries for impurity 1, impurity 2, impurity 3, and impurity 4 were calculated.

### Robustness

To determine the robustness of the developed method, experimental conditions were deliberately changed and the resolution between the TOLP, impurity 1, impurity 2, impurity 3, and impurity 4 were evaluated. The flow rate of the mobile phase was 1.0 ml/min. To study the effect of the flow rate on the developed method, 0.2 units of flow were changed (i.e. 0.8 and 1.2 ml/min). The effect of column temperature on the developed method was studied at 35 and 45°C (instead of 40°C). The effect of pH on the resolution of impurities was studied by varying ± 0.2 pH units (i.e. 7.8 and 8.2) and the mobile phase composition was changed ±10% from the initial composition. In all of the above varied conditions, the components of the mobile phase were held constant.

### Stability in solution and in the mobile phase

TOLP-spiked solutions (with respect to the specification, i.e., 0.5% level) were prepared in the diluent by leaving the test solutions at room temperature. The spiked solutions were injected at 0, 24, 48 hr time intervals. The impurity content was calculated, and the consistency in the % area of the principal peak at each interval was checked. The prepared mobile phase was kept constant during the study period. The mobile phase study was demonstrated by injecting the freshly prepared sample solution at different time intervals (0–2 days).

## Results and Discussion

### Optimization of chromatographic conditions

The main objective of the chromatographic method was to separate TOLP from its known impurities and degradants by reversed-phase chromatography. The separation of all impurities, including positional isomers, were tried using different mobile phases containing acetate buffers and phosphate buffers along with various ratios of organic modifiers like acetonitrile and methanol by using different gradient programmes. The impurities and degradants pertaining to the individual active moiety were estimated at the specific wavelength of 254 nm. Positional isomers were co-eluted, when different stationary phases such as C8, C18, phenyl, and cyano, along with different mobile phases were used. The pKa value of TOLP is 9.4. The separation of the positional isomers of TOLP was investigated using a phosphate buffer ranging from pH 5.0 to 9.0. The Inert Sustine 250 × 4.6, C18, 3 micron column was selected for wide pH compatibility, better separation, and diethylamine was used as a modifier to get better peak shapes. When pH 5.0 to 7.0 phosphate buffers (0.01M) were used in the gradient elution with acetonitrile, the positional isomers (TOLP and impurity 3) were eluted as a single peak. However, they were completely separated with good resolution (Rs > 2.0) when the phosphate buffers at pH 7.5–9.0 were used. This separation might be attributed to the difference in pKa values of positional isomers (TOLP and impurity 3). At pH 5.0 to 7.0, the polarities might be similar for TOLP and impurity 3, whereas at a pH above 7.5, the polarities might be different. Due to the difference in polarities at a pH above 7.5, separation between positional isomers (TOLP and impurity 3) had occurred. But at pH 9.0, a broad peak shape was observed for one of the positional isomers (Impurity 4).

Based on the above experimental data, the chromatographic separation was finalized by the following gradient program: time(min)/%B; T_0.01_,/50, T_5_/50, T_12_/60, T_15_/70, T_25_/90, T_30_/50, T_35_/50 by using a 0.01 M monobasic potassium phosphate buffer, pH adjusted to 8.0 with diethylamine. Mobile phase A consisted of a phosphate buffer and acetonitrile in the ratio of 50:50 (v/v). Mobile phase B consisted of a phosphate buffer and acetonitrile in the ratio of 30:70 (v/v). The flow rate of the mobile phase was 1.0 ml/min with a column temperature of 40°C and the detection wavelength at 254 nm. The injection volume was 10 μl. All of the impurities were well-separated with a resolution greater than 2.0, with typical retention times of TOLP, impurity 1, impurity 2, impurity 3, and impurity 4 being 17.224 min, 8.802 min, 10.701 min, 18.175 min, and 22.290 min, respectively.

### Response factor

The measurement of the response factor for each impurity determination is important when the calculations are being made on a relative percent basis. Hence, authentic samples of the known impurities and TOLP were dissolved in the diluent and injected, and then responses were calculated.

## Method Validation

Specificity, linearity, precision, accuracy, robustness, and ruggedness were done as part of the method’s validation.

### System suitability

Results from the system suitability study are given in Table 1.

**Fig. 3:**
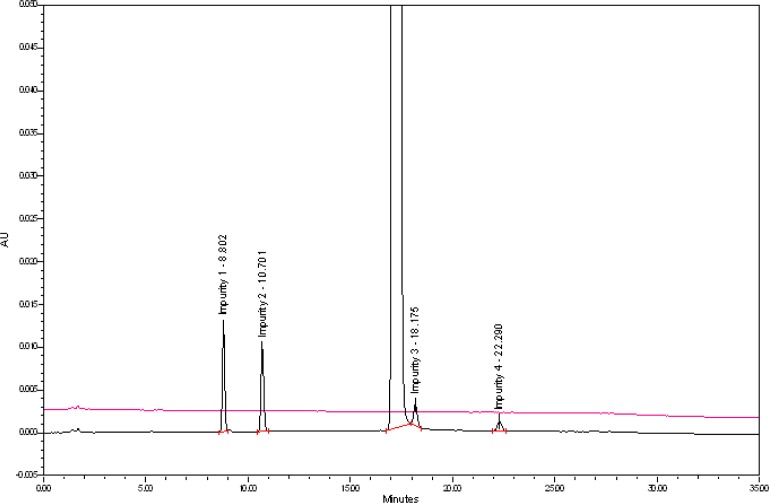
Typical overlay chromatogram of the blank and system suitability preparation

**Fig. 4:**
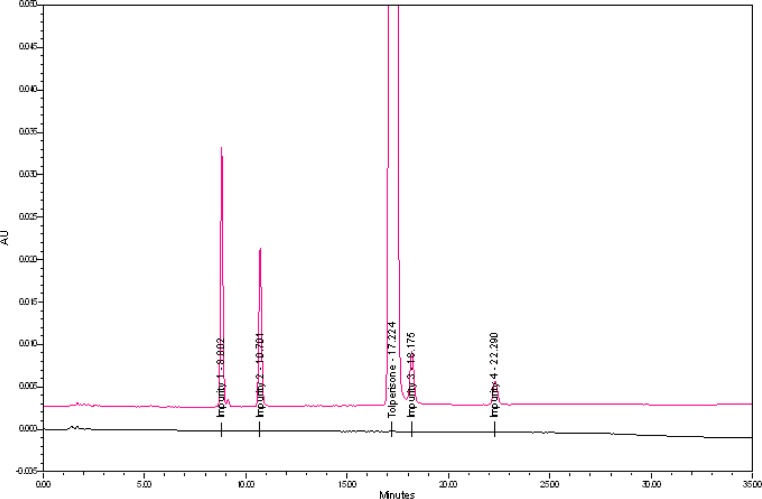
Typical overlay chromatogram of placebo and spiked test preparation

The typical overlay chromatogram of the blank and system suitability solution, placebo, and spiked test is shown in Fig. 3 & Fig. 4.

### Specificity – Stress study

All forced degradation samples were analyzed with the aforementioned HPLC conditions using a PDA detector to monitor the homogeneity and purity of the TOLP peak and its related impurities. The individual impurities, placebo, and TOLP were verified and proven to be non-interfering with each other, thus proving the specificity of the method.

No degradation was observed in the acid hydrolysis, heat stress, UV light, visible light, and humidity conditions, whereas significant degradation was observed in the base hydrolysis, water hydrolysis, and oxidative conditions. It is interesting to note that all of the peaks due to degradation were well-resolved and the degradation chromatograms are shown in Fig. 5A, 5B, & 5C. The chromatograms of the stressed samples were evaluated for peak purity of TOLP and its impurities using Waters Empower Networking Software. For all forced degradation samples, the purity angle (the weighted average of all spectral contrast angles calculated by comparing all spectra in the integrated peak against the peak apex spectrum) was found to be less than the threshold angle (the sum of the purity noise angle and solvent angle, the purity noise angles across the integrated peak), and there was no purity flag (the purity flag is an indication of spectral homogeneity, which compares the purity angle with the purity threshold) for the TOLP and its impurities. Also, the mass balance of the stressed samples was close to 100% (Table 2). This confirms the stability-indicating power of the developed method.

### Precision

The % RSD for the individual % of impurity 1, impurity 2, impurity 3, and impurity 4 in the impurities method precision study was within 2.5%. The results obtained in the intermediate precision study for the % RSD of the individual % of impurity 1, impurity 2, impurity 3, and impurity 4 were well within 1.6%, confirming the high precision of the method. The results are shown in Table 3.

### Limit of detection and quantification

The limit of detection of all impurities, namely impurity 1, impurity 2, impurity 3, and impurity 4, were achieved at 0.001%, 0.001%, 0.003%, and 0.006%. The limit of quantification for all impurities, namely impurity 1, impurity 2, impurity 3, and impurity 4, were achieved at 0.002%, 0.003%, 0.009%, and 0.019%. The % RSD of precision at the LOQ concentration for impurity 1, impurity 2, impurity 3, and impurity 4 was below 2.6%.

### Accuracy

The recovery of all of the four impurities from the finished dosage form ranged from 93.6 % to 110.7 %. The summary of the % recovery of the individual impurity is mentioned in Table 4.

### Linearity of response

The correlation coefficient obtained was greater than 0.999 for all of the related impurities of TOLP. The above results showed that an excellent correlation existed between the peak area and the concentration of impurity 1, impurity 2, impurity 3, and impurity 4. The linearity graphs for impurity 1, impurity 2, impurity 3, and impurity 4 are shown in Fig. 6A, 6B, 6C, & 6D.

### Robustness

In all of the deliberately varied chromatographic conditions (flow rate, column temperature, mobile phase composition, and pH variation), all of the analytes were adequately resolved, and the order of elution remained unchanged.

### Solution stability and Mobile phase stability

No significant changes were observed in the content of impurities, namely impurity 1, impurity 2, impurity 3, and impurity 4, during the solution stability and mobile phase stability experiments when performed using the impurities method. The solution stability and mobile phase stability experiment data confirmed that the sample solution and mobile phases used during the impurities’ determination was stable for at least 48 h.

## Conclusion

The gradient HPLC method developed for the determination of TOLP impurities in both the bulk drug and pharmaceutical dosage form was precise, accurate, and specific. The method was validated as per ICH guidelines and found to be specific, precise, linear, accurate, rugged, and robust. The developed method can be used for the stability analysis of the TOLP sample.

## Figures and Tables

**Fig. 5A:**
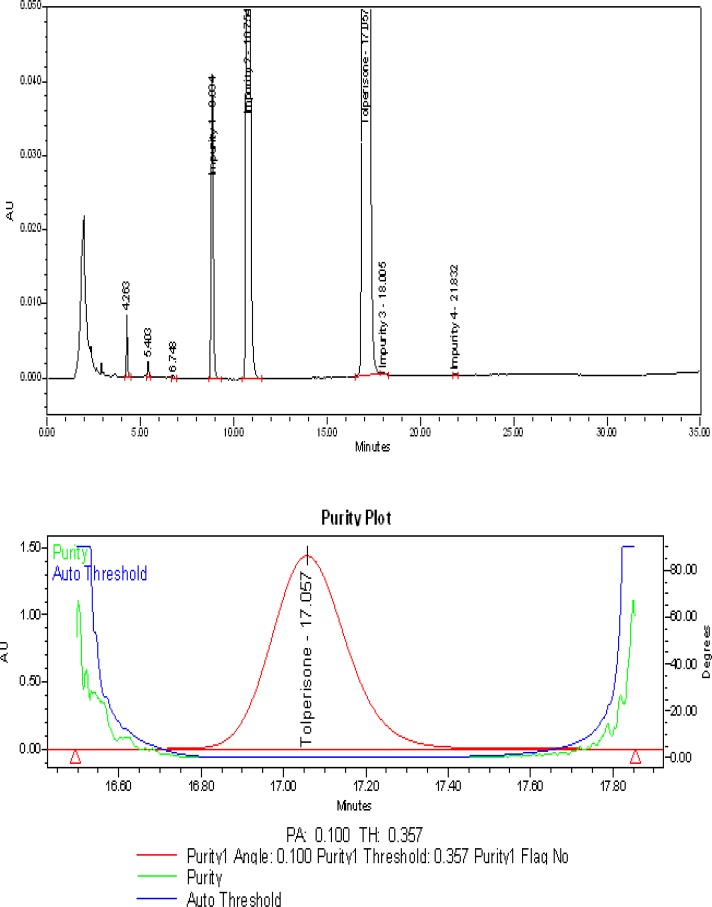
Typical chromatogram and purity plot of base stressed sample

**Fig. 5B:**
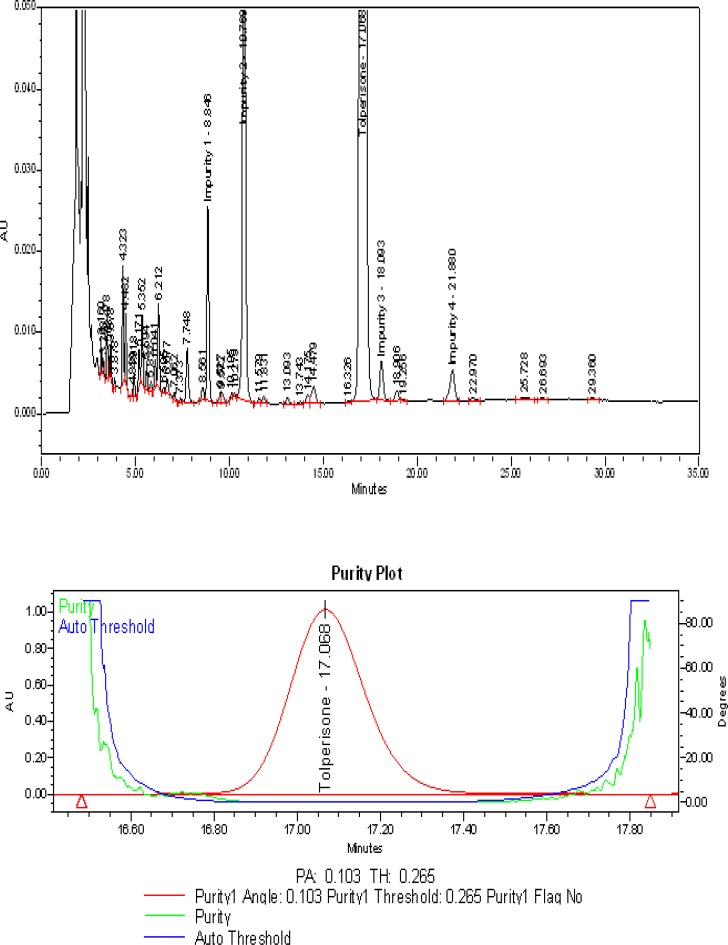
Typical chromatogram and purity plot of peroxide stressed sample

**Fig. 5C:**
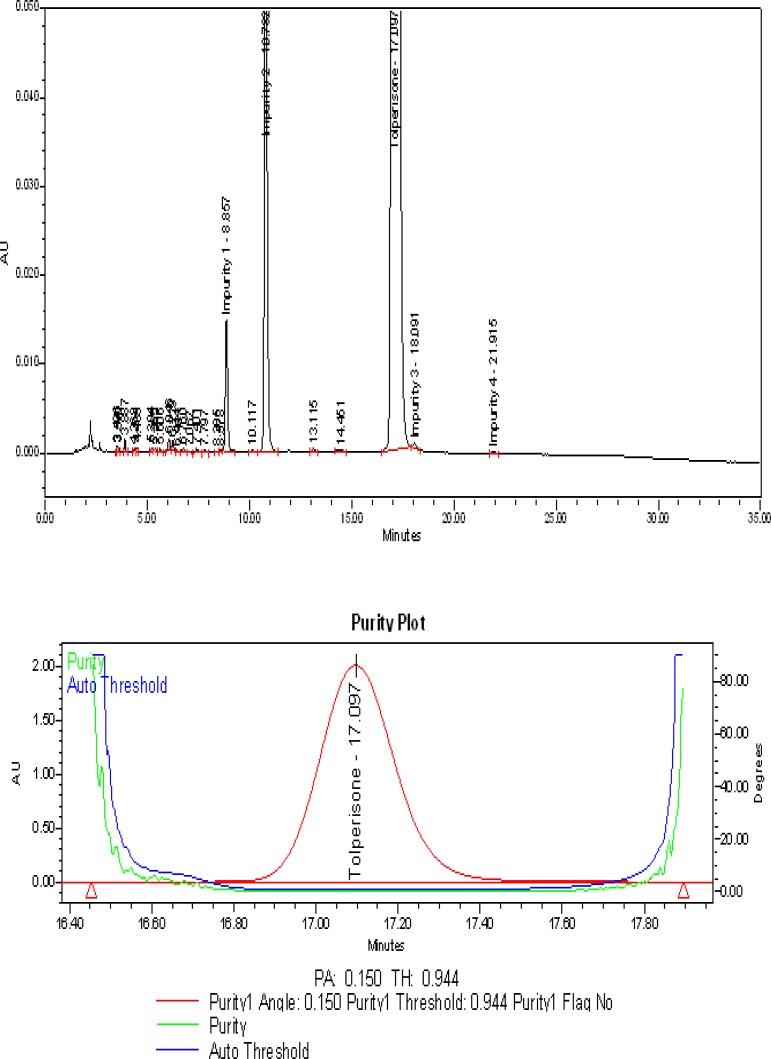
Typical chromatogram and purity plot of water stressed sample

**Fig. 6A:**
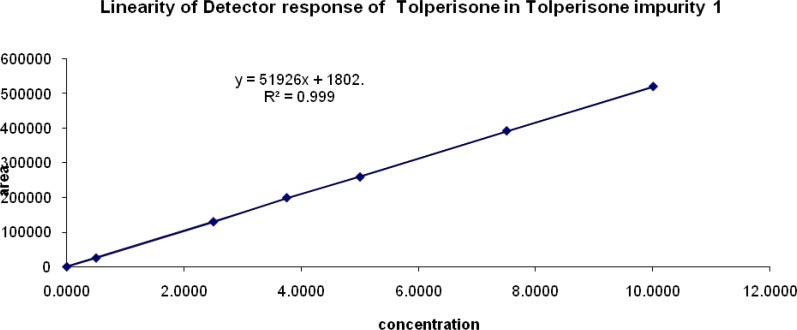
Linearity graph of impurity 1

**Fig. 6B:**
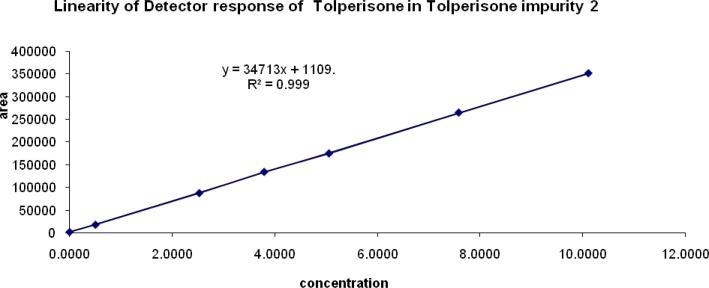
Linearity graph of impurity 2

**Fig. 6C:**
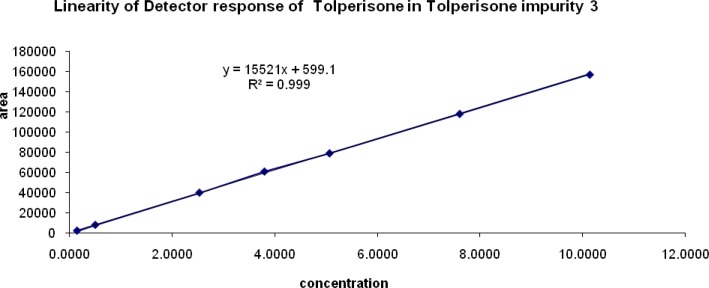
Linearity graph of impurity 3

**Fig. 6D:**
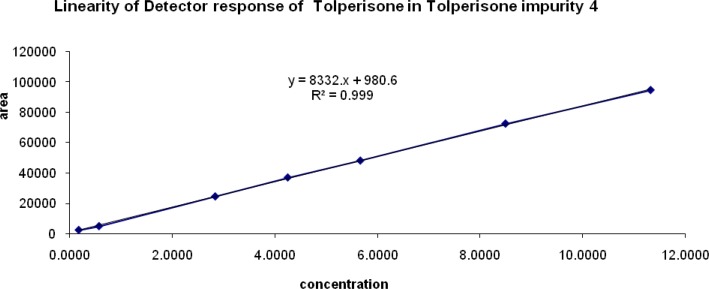
Linearity graph of impurity 4

**Tab. 1.** System suitability results

**System suitability parameters**	
Theoretical plates of Tolperisone	40148
Asymmetric factor for Tolperisone	1.1
Resolution between Tolperisone and impurity 3	2.7
Resolution between Tolperisone and impurity 2	21.6

**Tab. 2.** Forced degradation data for Tolperisone

**Degradation conditions**	**Tolperisone**			
	**Degradation %**	**Purity angle**	**Purity threshold**	**Mass balance %**
Refluxed with 0.1 N HCI solution for about 1 hr at 60 °C	0.1	0.222	1.078	99.2
Refluxed with 0.1 N NaOH solution for about 1 hr at 60 °C	20.0	0.100	0.357	100.2
Refluxed with 8% H_2_O_2_ solution for about 45 minutes at 60 °C	12.1	0.103	0.265	99.6
Exposed to light	0.2	0.134	1.025	100.1
Heated for about 2 hrs at 105 °C	0.2	0.082	0.628	99.8
Refluxed with purified water for about 90 minutes at 60 °C	2.8	0.150	0.944	99.1
Exposed to humidity at 25 °C, 90% RH for about 7 days	0.3	0.182	1.085	99.7

**Tab. 3.** Precision of the method

**Sample No.**	**% impurity**			
	**Impurity 1**	**Impurity 2**	**Impurity 3**	**Impurity 4**
1	0.457	0.501	0.466	0.508
2	0.470	0.515	0.479	0.519
3	0.471	0.517	0.486	0.540
4	0.473	0.521	0.484	0.536
5	0.467	0.514	0.476	0.519
6	0.486	0.535	0.495	0.539
Average	0.471	0.517	0.481	0.527
%RSD	2.0	2.1	2.0	2.5

**Tab. 4.** Accuracy of the method

**Spike level**	**% recovery^a^**			
	**Impurity 1**	**Impurity 2**	**Impurity 3**	**Impurity 4**
0.05% Level	96.9	110.7	93.6	96.8
0.25% Level	99.4	108.2	99.8	103.8
0.5% Level	98.4	106.3	101.0	103.4
0.75% Level	98.0	104.2	97.4	102.8
1.0% Level	97.8	104.4	97.7	101.5

a Mean for three determinations.
